# Neonatal Pain Assessment After Night Shifts in NICU Professionals: A Within‐Subject Exploratory Eye‐Tracking Study

**DOI:** 10.1155/ijpe/5596831

**Published:** 2026-10-10

**Authors:** Philipp Deindl, Alexandra Luister, Eik Vettorazzi, Johannes Mader, Nadine Pointner, Philipp Steinbauer, Johanna Alexopolus, Michael Wagner, Angelika Berger, Dominique Singer, Vito Giordano

**Affiliations:** ^1^ Department of Neonatology and Pediatric Intensive Care Medicine University Children′s Hospital University Medical Center Hamburg-Eppendorf Hamburg Germany uke.de; ^2^ Department of Pediatrics and Adolescent Medicine Hospital of Lüneburg Lüneburg Germany; ^3^ Department of Neonatology and Pediatric Intensive Care Medicine University Hospital Bonn Bonn Germany uni-bonn.de; ^4^ Institute of Medical Biometry and Epidemiology University Medical Center Hamburg-Eppendorf Hamburg Germany uke.de; ^5^ Department of Pediatrics and Adolescent Medicine Division of Neonatology Pediatric Intensive Care and Neuropediatrics Comprehensive Center for Pediatrics Medical University of Vienna Vienna Austria meduniwien.ac.at; ^6^ Center for Health and Bioresources Austrian Institute of Technology Vienna Austria ait.ac.at; ^7^ Department of Psychoanalysis and Psychotherapy Medical University Vienna Vienna Austria meduniwien.ac.at

**Keywords:** eye tracking, neonatal intensive care, neonatal pain, pupillometry, sleep

## Abstract

**Background:**

Night‐shift work may influence attention and emotion processing, potentially affecting clinical assessment. We investigated whether neonatal pain evaluation and associated visual‐attentional and pupillary responses differed between rested and post‐night‐shift assessments in experienced NICU professionals.

**Methods:**

In this prospective within‐subject study, 20 NICU professionals evaluated 42 standardized video clips of term neonates exposed to nonnoxious, short noxious, or prolonged noxious stimuli. Participants were assessed after at least 1 day off work and within 2 h after completing one or more consecutive night shifts. Pain ratings, eye‐tracking metrics, and pupil diameter were compared between conditions.

**Results:**

Overall pain ratings did not differ significantly between rested and post‐night‐shift assessments (3.56 ± 0.69 vs. 3.61 ± 0.72; *p* = 0.643; Cohen′s *d* = 0.11). Pupillary responses also showed no statistically significant differences. Exploratory eye‐tracking analyses suggested reduced visual exploration after night‐shift exposure, including fewer visits across several areas of interest and shorter fixation duration on the infant′s body. These secondary analyses were unadjusted for multiple comparisons and should therefore be considered hypothesis‐generating.

**Conclusions:**

No statistically significant differences in neonatal pain ratings or pupillary responses were detected following routine night‐shift work, whereas exploratory eye‐tracking measures suggested differences in visual‐attentional patterns. Given the small, selected sample and exploratory design, these findings do not establish equivalent or preserved performance between conditions. Larger prospective studies incorporating objective sleep measures and clinically relevant outcomes are warranted.

## 1. Introduction

Accurate pain assessment in neonates is a critical yet inherently challenging task for healthcare professionals. Newborns cannot verbalize discomfort, and clinicians must infer pain from subtle behavioral and physiological cues. Despite validated assessment tools and growing awareness of neonatal pain, inter‐rater variability remains considerable, particularly in high‐acuity environments such as neonatal intensive care units (NICUs) [1–4]. Such variability may arise not only from clinical inexperience but also from fluctuations in the cognitive and emotional state of the assessor [5–8].

Night‐shift work represents a potentially important yet underrecognized factor contributing to this inconsistency. Night shifts may involve sleep restriction, extended wakefulness, and circadian misalignment, all of which can influence cognitive and emotional functioning. NICU staff frequently work night shifts and extended hours in a high‐demanding environment [9, 10]. A robust body of evidence demonstrates that sleep loss impairs sustained attention, working memory, executive function, and emotional regulation. In medical contexts, fatigue has been associated with diagnostic errors, impaired decision‐making, and reduced empathic responsiveness [11–13]. These deficits are particularly concerning in neonatology, where timely interpretation of nonverbal cues is essential for effective care.

Eye‐tracking research has revealed that experienced professionals systematically focus on diagnostically relevant facial regions, particularly the mouth and brow, during infant pain evaluation, a pattern linked to greater rating accuracy [14–18]. At the same time, pupillometry has emerged as a sensitive marker of emotional arousal and empathic engagement, reflecting the affective resonance of the observer in response to perceived distress [16, 19–22].

Despite these insights, little is known about whether routine night‐shift work is associated with changes in the integrated perceptual, cognitive, and empathic processes underlying neonatal pain assessment. Prior work suggests that sleep loss slows visual orienting and reduces flexibility in gaze behavior [23–25], but its impact on the combined behavioral, attentional, and physiological components of neonatal pain evaluation has not been systematically studied.

To address this gap, we conducted a within‐subject study to examine whether neonatal pain assessment and associated physiological and visual‐attentional measures differ between rested and post‐night‐shift assessments in NICU professionals. We specifically examined whether post‐night‐shift assessment was associated with (1) differences in behavioral pain ratings, (2) differences in pupillary responses as an index of emotional arousal, and (3) changes in gaze patterns during the evaluation of neonatal pain expressions. Understanding whether these components remain stable or become vulnerable under real‐world fatigue conditions has important implications for clinical safety, staff well‐being, and training in neonatal intensive care.

## 2. Methods

### 2.1. Study Design and Setting

We conducted a prospective, within‐subject, pre‐post study to investigate differences in neonatal pain assessment among healthcare professionals between rested and post‐night‐shift conditions. The study was carried out at the Level IV Perinatal Center of the University Medical Center Hamburg‐Eppendorf (UKE), Germany. Ethical approval was obtained from the Ethics Committee of the Hamburg Medical Association (reference: PV7384). All procedures were conducted in accordance with the Declaration of Helsinki.

### 2.2. Video Stimuli

Video recordings of 14 healthy term neonates (Day 2 postpartum) undergoing routine capillary blood sampling were used as standardized stimuli. Three stimulus conditions were recorded per infant: a nonnoxious thermal stimulus (heel disinfection), a short noxious stimulus (heel prick using a SafetyLancet Neonatal, Sarstedt, Germany), and a prolonged noxious stimulus (heel squeezing for blood collection).

Filming was performed in a quiet room with the infant in a supine position using a GoPro Hero10 camera mounted above the torso. Audio was captured with a directional shotgun microphone (iGoMic Mini, MicW, China). Per local protocol, 30% glucose solution (0.1 mL/kg) was administered 1 min before the procedure. No pharmacological analgesia was used. Video clips were edited into 10 s segments using iMovie v10.3.8 (Apple Inc.), yielding 42 total clips (14 infants × 3 conditions), with half showing the full body and half facial close‐ups. The sequence of presentation was randomized via Tobii Pro Lab (v1.194, Tobii Technology, Sweden).

### 2.3. Participants

Healthcare professionals from the NICU at UKE were recruited between February and July 2024. Participants were tested twice. Preshift condition: after at least one full day off work. Postshift condition: within 2 h after completion of one or more consecutive night shifts. Participants completed questionnaires assessing usual nightly sleep duration (< 6, 6–8, and > 8 h), subjective sleep quality, and parental status.

### 2.4. Procedure

During both sessions, participants viewed all 42 randomized video clips on a 24‐inch monitor (1920 × 1080 px) under standardized lighting conditions. They rated the perceived pain intensity of each video on a 6‐point Visual Analogue Scale (VAS), ranging from 1 (“no pain”) to 6 (“maximum imaginable pain”). The VAS has previously shown reliability for observer‐based pain assessment in preverbal children [26–28].

### 2.5. Eye‐Tracking and Pupillometry

Visual attention and pupil size were recorded using a Tobii Pro Fusion eye tracker (sampling rate: 250 Hz), synchronized with Tobii Pro Lab software (Tobii AB, Danderyd, Sweden). A 5‐point calibration was conducted before each session. Defined Areas of Interest (AOIs) included the infant′s face (primary diagnostic zone), the heel (procedural site), and the background (nonrelevant area). Recorded metrics comprised total fixation duration within AOIs, fixation count, saccade frequency, and mean pupil diameter over time [6, 7, 29]. Stimulus onset markers were embedded for time‐locked analysis. All recordings were conducted under stable illumination to minimize light‐induced pupil variability. A simplified 6‐point VAS was implemented at the end of each video and presented randomly. This setting allowed rapid, both visual and button‐based responses within the Tobii Pro Lab environment, ensuring precise synchronization with eye‐tracking data.

### 2.6. Statistical Analysis

All statistical analyses were conducted in R (Version 4.3.3; R Foundation for Statistical Computing, Vienna, Austria). The primary endpoint was the difference in mean VAS pain ratings between the rested and post‐night‐shift conditions. Secondary endpoints included mean pupil diameter and eye‐tracking parameters (fixation duration, fixation count, saccade metrics, and early orienting latencies). To account for the repeated‐measures structure of the data and to avoid pseudoreplication, analyses were performed following a within‐subject pre‐post framework at the participant level. For each modality, measurements were first aggregated per participant (and, where applicable, across stimuli), and paired comparisons between rested and post‐night‐shift conditions were conducted using paired *t*‐tests. In addition to *p*‐values, results are reported as mean differences, Cohen′s d for paired samples, and corresponding 95% confidence intervals to provide estimates of effect magnitude and precision. This analytical approach was applied consistently across all modalities (VAS, pupillometry, and eye‐tracking parameters), and results were summarized in an integrated table to enhance comparability and transparency. Given the exploratory design and sample size, hypothesis testing followed a two‐sided alpha level of 0.05. No adjustment for multiple comparisons was applied to the secondary eye‐tracking outcomes. Accordingly, *p*‐values for these analyses are considered exploratory, and nominally significant findings should be interpreted as hypothesis‐generating rather than confirmatory. As an exploratory secondary analysis, a multivariable linear regression model was used to examine whether participant characteristics were associated with the change in VAS pain ratings between rested and post‐night‐shift assessments. Given the small sample size relative to the number of predictors, coefficient estimates from this model were considered exploratory and potentially unstable and were not intended for confirmatory or clinical inference. The regression included the mean change in VAS pain ratings between pre‐night‐shift and post‐night‐shift assessments (*Δ*VAS = pre − post) as the dependent variable. Independent variables included professional experience (years), professional category (nurses vs. physicians), and the number of previous night shifts completed by the participant. Regression coefficients, standard errors, and associated *p*‐values were calculated. All NICU personnel willing to participate within the predefined recruitment period were included. A post hoc power analysis was performed for the paired‐samples *t*‐test using a two‐sided *α* of 0.05 and an assumed medium effect size of Cohen^′^s d_z_ = 0.50. With a final paired sample of 20 participants, the estimated statistical power was 56.5%. This limited power indicates sensitivity to detect effects of moderate magnitude, and therefore supports cautious interpretation of nonsignificant findings. Accordingly, greater emphasis was placed on effect‐size estimates and corresponding 95% confidence intervals rather than on statistical significance alone.

## 3. Results

### 3.1. Participant Characteristics

Although 53 NICU/Intermediate Care (IMC) staff members were initially recruited and completed at least one rating session, only 26 participants were enrolled in the pre‐post‐night shift scenario. Six extra participants were excluded because of poor eye tracking data quality, leading to a complete pre‐post case scenario of a total of 20 participants. This drop in the sample size is mostly related to the fact that for this study, we focused basically on NICU staff. The dataset included 14 physicians and 6 nurses. The median age range was 31–35 years, and most participants reported between 0–10 years of professional experience. The majority identified as female. Usual sleep duration was most commonly reported as 6–8 h per night. Participants had completed a mean of 1.5 night shifts (SD = 0.8) in the 72 h preceding the second testing session. Comprehensive participant data are summarized in Table 1, and age and sleep categories are visualized in Figure 1.

**Table 1:** Participant characteristics: Variables are summarized as categorical distributions, reported in absolute numbers with corresponding percentages.

Variable	*n* (%)
Age (years)	
18–25	1 (5)
26–30	4 (20)
31–35	8 (40)
36–40	5 (25)
41–45	1 (5)
46–50	1 (5)
Profession	
Nurse	6 (30)
Physician	14 (70)
Professional experience (years)	
0–5	9 (45)
11–15	1 (5)
6–10	9 (45)
> 20	1 (5)
Gender	
Male	5 (25)
Female	15 (75)
Count of previous night shifts	
1	14 (70)
2	3 (15)
3	3 (15)
Usual sleep duration (hours)	
6–8 hours	18 (90)
> 8 hours	1 (5)
No information	1 (5)
Sleep quality	
No information	2 (10)
Bad	4 (20)
Good	6 (30)
Normal	8 (40)

**Figure 1:**
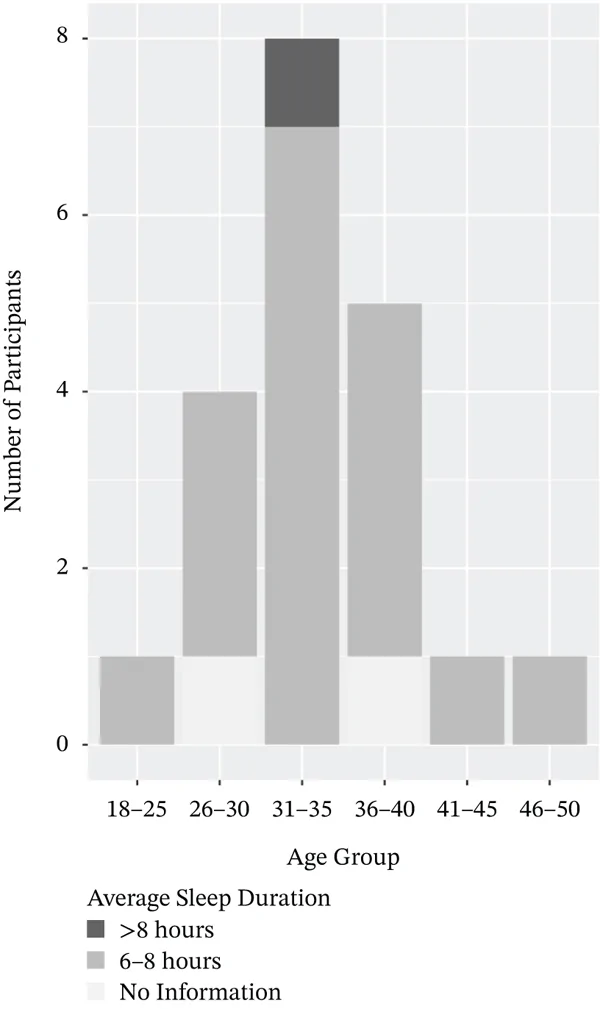
Age distribution and sleep‐related characteristics of NICU staff participants, grouped by usual sleep duration.

### 3.2. Pain Ratings Before and After Night Shifts

Overall pain ratings on the VAS remained comparable across pre‐night‐shift and post‐night‐shift assessments. Figure 2 illustrates individual trajectories and mean changes. Although when looking at global mean differences (all conditions), slight variations were observed; these differences were small in magnitude. Mean preshift ratings were 3.56 ± 0.69, compared with 3.61 ± 0.72 postshift, corresponding to a mean paired difference of 0.05. The paired *t*‐test yielded *p* = 0.643, with Cohen^′^s d = 0.11 [95% CI −0.34, 0.54]. Taken together, no statistically significant difference in overall subjective pain ratings was detected between rested and post‐night‐shift assessments. However, given the sample size and width of the confidence interval, this finding should not be interpreted as demonstrating equivalence between conditions.

**Figure 2:**
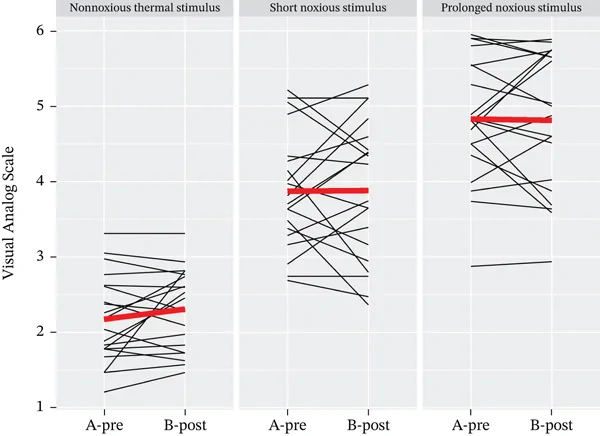
Individual pain ratings across three stimulus types (nonnoxious, short noxious, and prolonged noxious) (A) before and (B) after night shifts. Each line represents one participant; red lines indicate the group mean trajectory.

The rating on different kinds of stimulus within different numbers of previous night shifts is descriptively presented in Figure 3.

**Figure 3:**
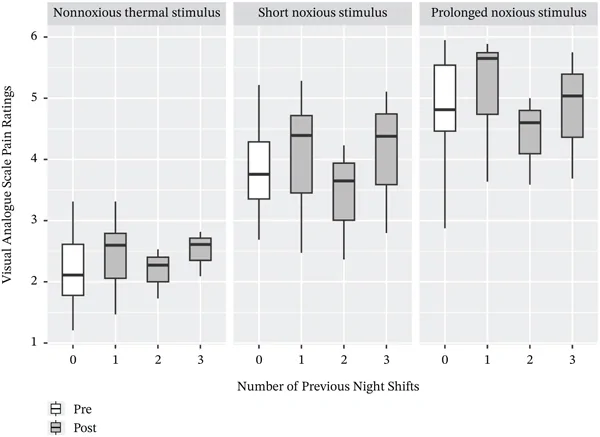
Distribution of pain ratings across categories of prior night shifts for each stimulus condition.

Moreover, a multivariable linear regression model was calculated to assess the association between participant characteristics and changes in VAS pain ratings between pre‐night‐shift and post‐night‐ shift assessments (*Δ*VAS = post − pre) accounting for the number of previous night shift (Figure 4). In the exploratory regression analysis examining factors associated with changes in pain ratings (*Δ*VAS), nurses demonstrated a significantly greater reduction in pain ratings following night shifts compared with physicians (*β* = −0.39, SE = 0.17, *p* = 0.030). This indicates that the decrease in pain assessment scores after night shift exposure was more pronounced among nursing staff. Neither professional experience (*β* = 0.02, *p* = 0.260) nor the number of previous night shifts (*β* = −0.04, *p* = 0.715) showed significant associations with changes in pain ratings. The overall regression model did not reach statistical significance (*F*(3, 47) = 1.96, *p* = 0.132) and explained a limited proportion of variance in rating changes (*R* ^2^ = 0.11, adjusted *R* ^2^ = 0.05).

**Figure 4:**
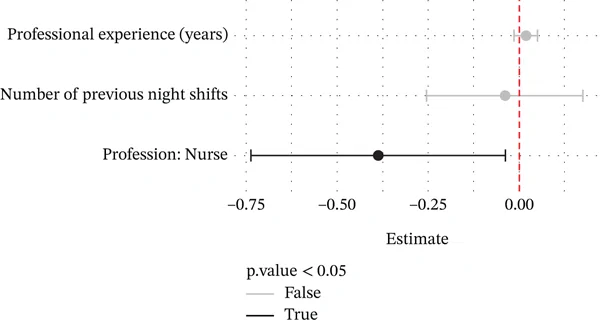
Forest plot of coefficients from the exploratory multivariable linear regression examining associations between participant characteristics and change in VAS pain ratings between rested and post‐night‐shift assessments.

### 3.3. Pupil Diameter Analysis

Pupillary responses remained largely unconditioned between the pre‐night‐shift and post‐night‐shift sessions. Figure 5 presents information on pupil diameter related to different type of stimuli, whereas Table 2 and Figure 6 provides information on pupil diameter related to different AOI. Descriptively, only minimal changes were observed across all AOI. Within the Face AOI, mean pupil diameter increased slightly from 3.57 ± 0.34 mm preshift to 3.61 ± 0.39 mm postshift, corresponding to a mean difference of 0.04 mm (*p* = 0.659, Cohen^′^s d = 0.10 [95% CI −0.34, 0.54]). Similarly, pupil diameter remained unchanged within the Body AOI (3.59 ± 0.35 vs. 3.60 ± 0.41 mm; mean difference = 0.01 mm; *p* = 0.939, Cohen^′^s d = 0.017 [95% CI −0.42, 0.45]) and the Surrounding AOI (3.68 ± 0.34 vs. 3.69 ± 0.39 mm; mean difference = 0.01 mm; *p* = 0.953, Cohen^′^s d = 0.01 [95% CI −0.42, 0.45]). Taken together, these findings indicate that pupillary responses were stable across conditions, with negligible effect sizes and no evidence of altered physiological arousal or emotional engagement following night‐shift exposure.

**Figure 5:**
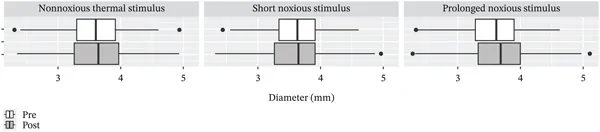
Boxplots of pupil diameter (in mm) before and after night shifts, stratified by stimulus type and camera perspective (facial close‐up vs. full‐body view).

**Table 2:** Multimodal pre–post comparison of eye‐tracking parameters and pupillometry divided by area of interest before and after night shifts.

AOI	Parameter	Mean pre	SD pre	Mean post	SD post	Mean difference	Cohen′s d	95% CI lower	95% CI upper	*p*
Body	Average fixation duration	262.709	50.517	237.697	53.451	−25.012	−0.509	−0.970	−0.036	0.035
Body	Number of fixations	2.394	0.527	2.349	0.607	−0.045	−0.090	−0.528	0.350	0.692
Body	Average pupil diameter	3.593	0.354	3.599	0.414	0.006	0.017	−0.421	0.455	0.939
Body	Time to entry saccade	4242.543	588.693	4267.392	517.772	24.849	0.048	−0.391	0.486	0.833
Body	Time to first visit	3121.424	362.551	3119.388	417.001	−2.036	−0.006	−0.444	0.432	0.979
Body	Number of visits	1.676	0.417	1.530	0.334	−0.146	−0.449	−0.904	0.017	0.059
Face	Average fixation duration	359.813	90.622	337.765	109.699	−22.047	−0.277	−0.720	0.173	0.231
Face	Number of fixations	5.621	1.142	5.730	1.545	0.109	0.087	−0.353	0.525	0.702
Face	Average pupil diameter	3.573	0.339	3.608	0.394	0.035	0.100	−0.340	0.538	0.659
Face	Time to entry saccade	2710.646	644.840	2855.408	829.999	144.763	0.178	−0.266	0.618	0.435
Face	Time to first visit	2261.930	342.717	2326.434	379.576	64.504	0.165	−0.279	0.604	0.470
Face	Number of visits	3.395	0.770	3.139	0.586	−0.255	−0.461	−0.917	0.006	0.053
Surrounding	Average fixation duration	300.415	55.663	292.517	57.244	−7.898	−0.110	−0.548	0.331	0.628
Surrounding	Number of fixations	4.394	1.962	3.861	1.537	−0.533	−0.282	−0.725	0.169	0.223
Surrounding	Average pupil diameter	3.687	0.343	3.692	0.396	0.005	0.013	−0.425	0.452	0.953
Surrounding	Time to entry saccade	3333.607	1258.537	3482.806	1310.128	149.199	0.106	−0.335	0.544	0.642
Surrounding	Time to first visit	2478.173	1130.679	2625.006	1184.513	146.834	0.173	−0.270	0.613	0.447
Surrounding	Number of visits	2.394	0.907	1.989	0.713	−0.406	−0.522	−0.984	−0.048	0.031

**Figure 6:**
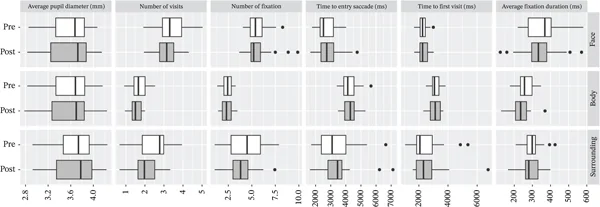
Eye‐tracking and pupillometry measures across predefined Areas of Interest (face, body, and surrounding) during rested and post‐night‐shift assessments.

### 3.4. Eye‐Tracking Metrics

Visual attention patterns directed at predefined AOIs showed consistent scanning behavior across pre‐night‐shift and post‐night‐shift sessions. Nevertheless, several eye‐tracking parameters suggested a tendency toward reduced visual exploration following night shift exposure (Table 2; Figure 6).

Within the Face AOI, average fixation duration decreased from 359.81 ± 90.62 ms preshift to 337.77 ± 109.70 ms postshift, corresponding to a mean difference of −22.05 ms. This difference was not statistically significant (*p* = 0.231, Cohen^′^s d = −0.27 [95% CI −0.72, 0.17]). The number of fixations remained similar (5.62 ± 1.14 vs. 5.73 ± 1.54; *p* = 0.702). Likewise, time to entry saccade (*p* = 0.435) and time to first visit (*p* = 0.470) also remained unchanged. However, the number of visits decreased from 3.39 ± 0.77 to 3.13 ± 0.58, corresponding to a moderate effect size (Cohen^′^s d = −0.46) and a trend toward statistical significance (*p* = 0.053).

Within the Body AOI, average fixation duration decreased significantly from 262.71 ± 50.52 ms preshift to 237.70 ± 53.45 ms post‐shift (mean difference − 25.01 ms; *p* = 0.035, Cohen^′^s d = −0.50 [95% CI −0.97, −0.036]). The number of fixations, time to entry saccade, and time to first visit remained unchanged. The number of visits showed a numerical reduction from 1.67 ± 0.41 to 1.53 ± 0.33 (*p* = 0.059, Cohen^′^s d = −0.44), suggesting a trend toward less frequent inspection of the procedural area after night shifts.

Within the Surrounding AOI, average fixation duration, fixation count, time to entry saccade, and time to first visit did not differ significantly between assessments. In contrast, the number of visits decreased significantly from 2.39 ± 0.90 preshift to 1.98 ± 0.71 postshift (mean difference − 0.40*p* = 0.031, Cohen^′^s d = −0.52 [95% CI −0.98, −0.04]).

Overall, most gaze and pupillary measures did not show statistically significant differences between assessments. Exploratory analyses identified a pattern of fewer visits across multiple AOIs and shorter fixation duration within the Body AOI following night‐shift exposure. However, because these secondary analyses involved multiple unadjusted comparisons, the observed nominally significant findings should be interpreted cautiously and considered hypothesis‐generating.

## 4. Discussion

This study examined whether neonatal pain assessment and associated attentional and pupillary measures differed between rested and post‐night‐shift assessments in NICU professionals. Participants maintained largely stable pain ratings and physiological indices of attention and arousal. In contrast to our previous mixed‐sample study including both NICU and IMC staff [16], this investigation focused exclusively on NICU specialists working in a high‐acuity environment requiring sustained vigilance and emotional attunement.

In this exploratory sample, we did not detect a statistically significant difference in neonatal pain ratings between rested and post‐night‐shift assessments. No statistically significant changes in pain ratings were detected following night‐shift exposure. However, eye‐tracking data revealed subtle changes suggestive of reduced visual exploration following night shifts, including fewer visits across multiple AOI and shorter fixation durations on the infant′s body region.

These findings are broadly in line with prior work linking sleep loss to subtle alterations in attentional processes and reduced flexibility in attentional allocation [30–32], whereas more automatized or learned aspects of visual processing appear relatively preserved. Together, this pattern suggests that routine night‐shift exposure may be associated with modest and selective differences in visual attention rather than a global disruption of perceptual or evaluative performance.

It is important to note that we did not measure clinical performance. Similar pain ratings do not necessarily imply unchanged bedside behavior: fatigue could still impair timing, communication, or motor precision in real care settings. In addition, subtle changes in visual exploration may not be fully captured by global rating measures alone. Future research should therefore include objective clinical outcomes alongside perceptual and physiological measures.

Overall, our findings suggest that experienced NICU professionals maintain stable neonatal pain assessments despite night‐shift exposure. However, eye‐tracking measures indicated a tendency toward reduced visual exploration, characterized by fewer visits to relevant visual regions and shorter fixation durations on the infant′s body. Although these changes did not translate into altered pain ratings, they may reflect subtle fatigue‐related adaptations in attentional processing. This pattern raises the possibility that clinical expertise preserves evaluative performance while allowing modifications in the visual strategies used to reach clinical judgments.

### 4.1. Impact of Night Shift Models on Cognitive and Empathic Performance

Beyond our own findings, prior work has shown that both cognitive vigilance and empathic responsiveness are highly sensitive to sleep loss and circadian disruption. Extended‐duration shifts (≥ 24 h) markedly increase error rates among residents [9, 10], and 12‐h nursing shifts have been associated with burnout and missed care [33, 34]. Shift design is therefore crucial: forward‐rotating, rapidly cycling schedules better support alertness and recovery than backward or slow rotations [35, 36], whereas “quick returns” (< 11 h between shifts) further heighten fatigue and injury risk [37–39]. Current recommendations accordingly suggest limiting consecutive night shifts to no more than two or three [37, 40]. This was in accordance with our results where the maximum number of previous night shifts was not more than three consecutives. Following on this point, our regression analysis did not show an effect of number previous night shifts on pain rating, confirming previously mentioned literature on the topic.

Sleep deprivation has also been shown to impair emotion recognition and prosocial behavior [12, 41], with studies in medical trainees linking fatigue to reduced empathic responsiveness [42]. At the same time, some studies report relatively stable performance in pain recognition across shifts, albeit with more subtle changes in sensitivity. For example, Schmidt et al. [43], observed stable pain intensity ratings alongside shifts in perceptual sensitivity over the course of a shift, suggesting a more nuanced pattern rather than complete stability. Although mitigation strategies such as scheduled naps and circadian lighting may help reduce fatigue‐related impairments reported in other healthcare settings, our findings suggest that neonatal pain assessment performance remained preserved following night shift exposure in this cohort. [44].

### 4.2. Limitations

This single‐center exploratory study has several important limitations that should be considered when interpreting the findings. First, the sample size was small, with complete paired data available for only 20 participants, which limits statistical power to detect small‐to‐moderate effects. As a result, the absence of large or consistent differences between conditions should not be interpreted as evidence of no effect, but rather as an absence of strong detectable effects under the present study conditions. Second, the study did not directly quantify sleep loss associated with night‐shift work. Actual sleep duration before testing was not recorded, and objective sleep measures such as actigraphy or polysomnography were not obtained. Consequently, we cannot determine whether individual participants were sleep‐deprived or quantify the degree of fatigue or sleep loss at the time of assessment. The exposure investigated in this study should therefore be interpreted as routine night‐shift work rather than experimentally or objectively confirmed sleep deprivation. Although subjective sleepiness was assessed, the manipulation may not have been sufficient to elicit measurable impairments across all domains. It remains possible that more pronounced effects would emerge under conditions of greater cumulative sleep loss or circadian disruption. Third, there was a substantial reduction from the initially assessed cohort to the final complete‐case sample. Importantly, this reduction reflects both noncompletion of the paired assessment protocol and subsequent data‐quality exclusions, and should therefore not be interpreted solely as conventional longitudinal attrition. Nevertheless, the resulting complete‐case sample may represent a selected subgroup of the initially assessed staff. Participants who completed both assessments and provided sufficient eye‐tracking data may differ systematically from noncompleters with respect to factors such as scheduling flexibility, motivation, tolerance of night‐shift work, or other unmeasured characteristics. This potential selection bias limits the external validity and generalizability of the findings to the broader population of NICU professionals. Fourth, the administration of 30% glucose prior to procedures, although ethically appropriate, may have attenuated neonatal pain expressions in the stimuli, and thereby reduced variability in observed responses. Furthermore, multiple secondary eye‐tracking outcomes were examined without adjustment for multiple comparisons. Consequently, the nominally significant findings may reflect Type I error and would not remain statistically significant under conventional multiplicity correction. These results should therefore be considered exploratory and hypothesis‐generating, and require confirmation in studies with prespecified eye‐tracking endpoints. Finally, the study focused on perceptual, attentional, and physiological markers using standardized video‐based assessments and did not include direct measures of clinical performance in real‐world settings. Consequently, the extent to which these findings translate to bedside decision‐making or patient outcomes remains uncertain.

### 4.3. Implications and Future Directions

In this exploratory study, no statistically significant differences in behavioral pain ratings or pupillary responses were detected between rested and post‐night‐shift assessments. These findings indicate that large differences were not evident in this sample, but they do not establish equivalent performance between conditions. Behavioral pain ratings and pupillary responses showed no evidence of substantial fatigue‐related impairment, supporting the notion that clinical expertise may help maintain core evaluative functions under conditions of moderate sleep restriction.

At the same time, eye‐tracking analyses revealed a consistent pattern of reduced visual exploration, characterized by fewer visits across multiple AOI and shorter fixation durations on the infant′s body region. Although these effects were generally small to moderate in magnitude, they may indicate subtle shifts in attentional strategies following night shifts. Rather than reflecting impaired performance, such changes could represent a more selective or efficient allocation of visual resources under fatigue.

Future studies should investigate whether these alterations in visual exploration are adaptive or potentially associated with reduced sensitivity to clinically relevant cues. Larger multicenter studies incorporating objective sleep measures, simulation‐based assessments, and real‐world clinical outcomes will be essential to determine whether these subtle attentional changes translate into meaningful effects on neonatal care and decision‐making.

## Author Contributions

P.D. conceived the study, designed the protocol, and supervised the project. V.G., A.L., P.S., N.P., J.M., J.A., and M.W. collected data and prepared database. E.V. and P.D. performed statistical analyses. P.D., A.L., A.B., D.S., and V.G. interpreted the results. P.D. and V.G. drafted the first version of the manuscript. A.B., M.W., D.S. critically reviewed the manuscript.

## Funding

This study was funded by the Medizinische Universität Wien (10.13039/501100005788).

## Disclosure

All scientific content, analyses, and interpretations remain the sole responsibility of the authors, who reviewed and approved the final version of the manuscript. All authors approved the final version of the manuscript.

## Ethics Statement

The present study was conducted in accordance with institutional and national guidelines. Ethical approval was obtained from the Ethics Committee of the Hamburg Medical Association (reference: PV7384). All procedures were conducted in accordance with the Declaration of Helsinki.

## Conflicts of Interest

The authors have nothing to report.

## Data Availability

The data that support the findings of this study are available from the corresponding author upon reasonable request.
